# To the Editors of the Medical and Physical Journal

**Published:** 1805-01-01

**Authors:** John Whitlam

**Affiliations:** Nottingham


### *To the Editors of the Medical and Physical Journal.*

GENTLEMEN,

WHEN we can snatch a fellow-creature from impending death, or even alleviate his sufferings, and render the remaining period of his existence comfortable, we feel the cheering influence of a ray emanating from the purest source of pleasure. Our gratification is more heightened, if the means made use of promise to contribute to the relief of others labouring under similar afflictions.

In compliance with the request of Mr. Jenkinson, in the 64th Number of your Journal, I shall state the strikingly beneficial effects of the solutio arsenici in an inveterate case of chronic rheumatism.

——— Ward, a female, who is now about the age of 35, was suddenly attacked, about two years since, with a violent pain near the middle of the spine, which hindered her from bending forwards without extreme difficulty. It was accompanied by a distressing pain in the epigastric region, attended by dyspepsia. She was pregnant at that time, and in a few days a miscarriage ensued. The pain then nearly left her; but it returned with redoubled violence in about a month. Her joints became affected, particularly her knees and ankles, which rendered walking extremely irksome; those of her fingers began to swell, and one of them became enlarged to nearly twice its proper size. She received only a temporary alleviation by the aid of remedies usually applied in such cases, though assisted by electricity.

She gradually became worse till the month of June last, when she began to take the solution, as recommended by Mr. J. In a few weeks she was much relieved, and was capable of walking without being incommoded by the pain; and since that time the swelling of her joints has decreased, and her general health is much improved.

There are two things worthy of particular notice in this case. 1st. The disease never put on the characteristic appearance of acute rheumatism. 2d. From the peculiar appearance of the countenance, and state of the pulse, tonic remedies seemed indicated; but none of them produced the effect of invigorating the system till arsenic was given. She still perseveres in using the medicine, and has the very pleasing prospect of a permanent cure from the use of it. I am, &c.

*Nottingham, Nov. 7, 1804.* JOHN WHITLAM.

